# The Phenylpropanoid Pathway Is a Central Roundabout in Peach Fruit Pre- and Postharvest Physiology

**DOI:** 10.3390/metabo16030191

**Published:** 2026-03-12

**Authors:** Lorena Melet, Ricardo Nilo-Poyanco, Maria Paz Covarrubias, Reinaldo Campos-Vargas, María Luisa Valenzuela, Andrea Miyasaka Almeida

**Affiliations:** 1 Centro de Genómica y Bioinformática, Facultad de Ciencias, Ingeniería y Tecnología, Universidad Mayor, Camino La Pirámide 5750, Huechuraba 8580745, Santiago, Chile; lorena.melet@mayor.cl; 2 Millennium Science Initiative Program, Millennium Institute for Integrative Biology (iBio), Santiago 7500565, Chile; 3 Millennium Science Initiative Program, Millennium Nucleus in Data Science for Plant Resilience (Phytolearning), Santiago 8370186, Chile; 4 Escuela de Biotecnología, Facultad de Ciencias, Ingeniería y Tecnología, Universidad Mayor, Camino La Pirámide 5750, Huechuraba 8580745, Santiago, Chile; ricardo.nilo@umayor.cl; 5 Departamento de Fruticultura y Enología, Facultad de Agronomía y Sistemas Naturales, Pontificia Universidad Católica de Chile, Santiago 8331150, Chile; mariapaz.covarrubias@gmail.com; 6 Facultad de Medicina Veterinaria y Agronomía, Universidad de Las Américas, Sede Providencia, Manuel Montt 948, Santiago 7500000, Chile; 7 Departamento de Producción Agrícola, Universidad de Chile, Santiago 8820808, Chile; reinaldocampos@uchile.cl; 8 Grupo de Investigación en Energía y Procesos Sustentables, Instituto de Ciencias Aplicadas, Facultad de Ingeniería, Universidad Autónoma de Chile, Campus El Llano, Ricardo Morales 3319, San Miguel 89110339, Santiago, Chile; maria.valenzuela@uautonoma.cl; 9 Escuela de Agronomía, Facultad de Ciencias, Ingeniería y Tecnología, Universidad Mayor, Camino La Pirámide 5750, Huechuraba 8580745, Santiago, Chile

**Keywords:** cold storage, *Prunus persica*, quinic acid, ripening, thinning

## Abstract

Background: Peach fruit quality can be compromised by cold storage, a postharvest practice required for long-distance export that can trigger chilling injury and metabolic disturbances affecting sugars, organic acids, and other metabolites. Preharvest practices such as thinning modify source–sink relationships and fruit development, potentially influencing susceptibility to chilling stress. Objectives: This study aimed to determine whether commercial thinning alters fruit susceptibility to cold storage damage and to identify metabolic processes associated with chilling tolerance in two nectarine varieties with contrasting sensitivity, ‘Magique’ (tolerant) and ‘Red Pearl’ (sensitive). Methods: Fruits from thinned (TH) and unthinned (UTH) trees were subjected to cold storage (0 °C, 21 days) followed by ripening, and evaluated for physiological parameters, sugar and organic acid composition by HPLC, and phenylpropanoid-related metabolites by ^1^H-NMR. A genome-scale metabolic model was built to model fruit metabolism using COBRApy. Results: Thinning increased fruit size in both varieties. Magique exhibited overall metabolic stability across thinning treatments and cold storage. Red Pearl, in contrast, showed broad metabolic fluctuation in response to external stimuli. Integration of transcriptomic data and metabolic modeling identified quinate-centered reactions as candidate regulatory nodes associated with phenylpropanoid flux during ripening and post-chilling recovery. Conclusions: These findings indicate that modulating quinate metabolism during early ripening may help improve chilling tolerance and highlight the phenylpropanoid pathway as a central metabolic axis modulated by both pre- and postharvest practices, with implications for fruit quality management.

## 1. Introduction

*Prunus persica* (L.) Batsch (peaches and nectarines) is a major stone fruit crop worldwide, with great economic relevance for Chile, which has ranked among the top ten *P. persica* exporters over the past five years (FAOSTAT, “Export Quantity”) [1]. However, consumer acceptance in overseas markets has declined in recent years due to reduced fruit quality. This problem is particularly pronounced in Chilean exports, as extended postharvest cold storage is required to ensure that fruit reaches distant markets in an acceptable condition [2,3]. Although peach and nectarine fruits are highly perishable and cold storage is commonly used to extend shelf-life, this practice can negatively affect *P. persica* fruit quality [4].

Consumer acceptance of peaches and nectarines in foreign markets depends on meeting specific quality standards, which include sensory and mechanical attributes, particularly taste and texture [4,5]. Among the metabolites that directly influence *P. persica* fruit taste, sugars and organic acids play a central role [6,7]. Texture is largely determined by mesocarp tissue structure, cell wall architecture, and intercellular space characteristics, all of which are modified during fruit ripening, a developmental process that renders the fruit edible [8,9]. Although these traits are genotype-dependent, several preharvest factors, such as weather conditions, growing season, rootstock, and agronomical practices, can significantly influence final fruit quality [4]. Therefore, the implementation of appropriate preharvest treatments may represent a promising strategy to improve fruit quality.

*P. persica* fruit quality impairment due to cold storage, commonly referred to as chilling injury (CI), is a complex physiological disorder. Exposure to low temperatures triggers extensive transcriptomic reprogramming [10], alters membrane lipid composition, and affects mesocarp texture and color [1,11,12], along with significant proteomic and metabolomic changes [13], among other alterations. One of the phenotypes associated with CI is fruit flesh bleeding (FBL), also known as internal reddening, which is characterized by the diffusion of anthocyanin pigments normally restricted to the pit cavity into the surrounding mesocarp tissue [13,14]. Not all cultivars develop FBL after cold storage, and when present, this phenotype does not necessarily compromise other organoleptic attributes of the fruit.

To prevent or mitigate disorders triggered by CI in *P. persica* fruit, several postharvest strategies have been evaluated, including heat treatment [15], controlled atmosphere storage [16], preconditioning [17], and applications of melatonin [18,19] or glycine betaine [20]. These approaches encompass both physical and chemical interventions aimed at limiting CI-induced quality deterioration. In contrast, preharvest strategies may offer a more integrative and potentially more effective approach, as they not only help preserve fruit quality during cold storage but can also enhance intrinsic fruit attributes [4,21]. To date, preharvest efforts to reduce CI have combined orchard management practices with hormonal treatments as part of a comprehensive fruit preservation strategy. These include applications of gibberellins [22,23], oxalic acid [24] and aminoethoxyvinylglycine [25]. However, crop load management emerges as an additional strategy with the potential to influence CI susceptibility. Among preharvest practices that control tree crop loading, fruit thinning represents a promising approach.

Thinning is a preharvest practice commonly used to enhance fruit quality in terms of size and total soluble solids (TSS) content [26,27,28,29]. It consists of the removal of fruits at the beginning of development to favor the growth of the remaining fruits. Thinning has also been shown to affect fruit metabolite composition during development by driving resources to specific pathways, such as the phenylpropanoid pathway, impacting final fruit quality [28,29,30].

While thinning is known to improve fruit size, TSS content and metabolite composition [28], its influence on fruit susceptibility to CI remains unclear. In this work, we analyzed primary metabolite profiles of nectarine fruits cv. ‘Magique’ and ‘Red Pearl’, known to possess different susceptibilities to CI [10], ripened at room temperature or after cold storage at 0 °C for 21 days, from trees subjected to different thinning treatments. Harvest and ripening quality parameters such as fruit weight, firmness, acidity and TSS content were measured. In addition, public RNA-seq data [10] were used to assess gene expression in regular and cold-stored ripe fruit from both varieties. Using these data, we aimed to evaluate whether the preharvest strategy of tree thinning could alter fruit susceptibility to CI.

## 2. Materials and Methods

### 2.1. Plant Materials and Experimental Design

**Figure 1:**
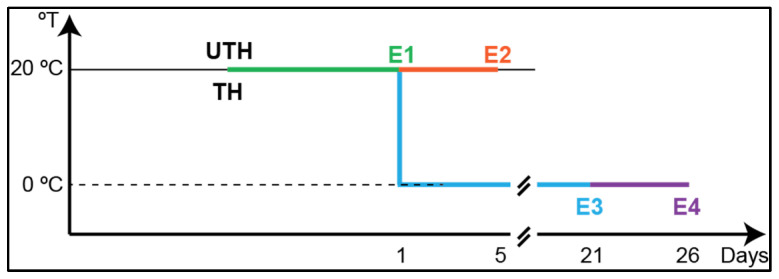
Schematic representation of postharvest conditions under evaluation in this study. Fruits were harvested with 57.7 N of firmness from unthinned (UTH) and thinned (TH) trees at harvest (E1, green). Three groups of 18 fruits each were subjected to different postharvest conditions after harvest (E1): 5 d at 20 °C (E2, shelf-life; orange), 21 d at 0 °C (E3, blue), and 21 d at 0 °C followed by 5 d at 20 °C (E4, purple).

Assays were performed with nectarine fruits (*P. persica* (L.) Batsch) of two different varieties grown in a commercial orchard in El Tambo, in the O’Higgins Region of Chile (34°28′30.4″ S, 70°59′07.5″ W), during the growing season of September 2014 until February 2015. The varieties selected were Magique (MG), an early-season variety, and Red Pearl (RP), a late-season variety, harvested 34 days later than MG. Regulation of the fruit load was carried out in two trees per treatment by manual thinning (34 leaves per fruit, commercial thinning, TH) and unthinned control trees with no fruit removed manually (10 leaves per fruit, UTH). Thinning was performed at 40 days after full bloom (DAB) for the MG variety and at 47 DAB for the RP variety. Fruits were handpicked at the commercial harvest stage, with a flesh firmness of 57.0 N at 122 DAB for MG and 61.2 N at 156 DAB for RP. Fruits were selected based on a uniform color and size and an absence of visual defects and divided into four developmental groups. The first group (E1) corresponded to the harvest stage; the second group (E2, shelf-life) was ripened at 20 °C for 5 days; the third group (E3, cold-stored fruit) was stored at 0 °C for 21 days; and the fourth group (E4, cold-stored plus shelf-life) was stored at 0 °C for 21 days, followed by 5 days at 20 °C (Figure 1). Fruit mesocarp was cut, frozen in liquid nitrogen, and stored at −80 °C for further analysis. For each group and variety, three to five biological replicates, each obtained from different fruits, were prepared. There is no data for E3 unthinned trees of the RP variety because the fruits were accidentally frozen during cold storage.

Physiological parameters were measured in 18 fruits per variety at the harvest (E1), shelf-life (E2), cold-stored (E3), and cold-stored plus shelf-life (E4) stages. The fruits were weighed, and equatorial diameter was measured using a digital caliper. Firmness was measured on two opposite sides of each fruit using a penetrometer fitted with an 8 mm diameter plunger. Titratable acidity was measured in 5 mL of fruit juice by the addition of 0.1 N NaOH until a pH of 8.2 was reached, and the NaOH volume was recorded to calculate titratable acidity expressed as a percentage of malic acid. Finally, the fruit juice percentage was calculated by paper absorption of the free juice from fruit belonging to groups E2 and E4, as previously described [31].

### 2.2. Lignin Staining

To determine the onset of pit hardening, specific lignin staining was performed using phloroglucinol [32] in a selection of representative fruits. Transverse and longitudinal sections were made from three fruits, and a solution of 5% phloroglucinol in 85% ethanol was added to the stone. The excess was removed, and fuming HCl was added to induce a magenta coloration in lignified mesocarp tissue. The samples were finally washed with 95% ethanol. Next, samples were visually inspected to evaluate the mesocarp staining.

### 2.3. Extraction of Polar Metabolites

Frozen fruit and leaf samples were ground with a liquid nitrogen-chilled analytical mill (IKA^®^ A11 basic) into a homogeneous powder of <0.15 mm particle size before lyophilization. The hydroalcoholic extraction of polar metabolites was performed using a protocol described by Moing et al. [33], using 30 mg of each lyophilized powder sample.

### 2.4. Liquid Chromatographic Analysis of Sugars and Organic Acids

Major soluble sugars and organic acids were measured in three to five replicates of each variety at each developmental stage (E1–E4) using liquid chromatography coupled with pulsed amperometric detection (PAD) or diode array detection (DAD). Sugars were measured in a HPAEC-PAD (Dionex DX-500, Sunnyvale, CA, USA) equipped with an autosampler (AS50), pump system (GP50) and electrochemical detector (ED40), and two tandem columns, CarboPac PA1 (250 × 4.5 mm, 5 μm; ThermoFisher, Waltham, MA, USA) at 40 °C using 200 mM NaOH and ultrapure (MilliQ^®^) water for the separation of each component. The following analytical conditions were used: flow rate 1.5 mL min^−1^ in reversed phase, eluent NaOH 200 mM, loop size of 25 µL and injection volume of 150 µL. Organic acids were measured by a HPLC-DAD (Jasco, Tokyo, Japan) equipped with a quaternary gradient pump system (Jasco PU-2089s plus) and a diode array detector (Jasco MD-2010 plus) using an Inertsil ODS-3 (C18) column (250 × 4.5 mm, 5 μm; GL Sciences, Shanghai, China) at 21 °C. The following analytical conditions were used: flow rate ramp from 0.7 to 1.7 mL min^−1^ in reverse phase, eluent KH_2_PO_4_ 50 mM pH 2.4, loop size 20 µL and injection volume 50 µL with absorbance detection at 212 nm. The results were analyzed using a calibration curve for each major sugar (fructose, myo-inositol, glucose, sorbitol, and sucrose) and major organic acid (malic acid, quinic acid, and citric acid) (Figures S1 and S2). The results were normalized by dry weight.

### 2.5. 1D ^1^H-NMR Targeted Profiling

Proton nuclear magnetic resonance spectroscopy (^1^H-NMR) quantitative profiling was used to measure phenylalanine, quinate, and caffeoylquinate in RP fruits at E1 for TH and UTH conditions. Data from MG fruits were obtained from previous work [28]. This was performed as described previously [28,34]. pH-adjusted extracts prepared in 200 mM deuterated phosphate buffer were analyzed using a 500 MHz Avance III spectrometer (Bruker, Wissembourg, France) equipped with a BBI 5 mm probe (Bruker). The ERETIC method was used to quantify absolute concentrations of all identified metabolites with three calibration curves using AMIX Bruker software (v. 3.9.14). The metabolite concentrations in each NMR tube and the metabolite contents in fruit were calculated using AMIX and Excel (Microsoft, Redmond, WA, USA).

### 2.6. Peach Fruit Metabolic Model

A genome-scale metabolic model (GSM) of *P. persica* fruit metabolism was constructed to represent the ripe fruit as a heterotrophic sink organ, with a specific focus on metabolic processes associated with ripening quality traits. The model was designed to capture the coordinated biosynthesis of ethylene, phenylpropanoids, anthocyanins, and cell wall degradation products, while maintaining cellular energy and redox homeostasis. Pathways and associated genes were obtained from PPERSICACYC, available through the Plant Metabolic Network portal [35] and from the specialized literature [34,36,37,38].

The model represents a steady-state, non-growing tissue and therefore does not include a biomass objective. Instead, cellular viability is enforced via a non-growth-associated maintenance (NGAM) ATP demand. Internal metabolite pools and stored reserves are explicitly represented to reflect the physiological context of a ripe fruit relying on remobilization of accumulated substrates.

The metabolic network was assembled from a curated reaction list stored in a tab-delimited file (Table S3). Each reaction entry includes a unique reaction identifier, a stoichiometrically balanced equation using compartment-specific metabolite identifiers (cytosolic (_c), mitochondrial (_m), and vacuolar (_v)), and (where available) a gene–protein–reaction (GPR) association based on the Ppersica_298_v2.1 database from the Plant Metabolic Network (PMN). Metabolic and transport reactions were manually inspected and loaded programmatically using COBRApy [39].

To prevent thermodynamically unrealistic solutions while allowing internal flexibility, flux constraints were applied to internal pool reactions for key cofactors (e.g., ATP, NAD(P)(H), CoA). These reactions allowed for the mobilization of intracellular reserves up to a defined upper bound. To simulate the minimal energetic requirements of ripe fruit, the non-growth-associated maintenance (NGAM) reaction was assigned a strictly positive lower bound (1.0 mmol·gDW^−1^·h^−1^). To avoid bypassing central carbon metabolism, fluxes for citrate and chlorogenate mobilization were constrained to zero, ensuring the model relied on *de novo* synthesis or alternative substrates where specified.

Rather than maximizing biomass, the model optimizes a custom ripening quality objective, defined as a pseudo-reaction called “RIPENING_QUALITY_SINK” (RQS):

*chlorogenate_c + cyanidin_c + ethylene_c + galacturonate_c → ∅*

Maximizing this reaction enforces the simultaneous production of four major ripening-associated traits: chlorogenate (phenylpropanoid metabolism), cyanidin (anthocyanin biosynthesis), ethylene (climacteric hormone), and galacturonate (pectin depolymerization proxy).

Flux balance analysis (FBA) was performed using COBRApy with default linear programming solvers. Model feasibility and pathway functionality were tested through a combination of objective maximization, targeted reaction forcing (lower-bound constraints), metabolite demand/sink diagnostics, and incremental network repair guided by stoichiometric consistency. All reported solutions correspond to optimal steady-state flux distributions.

Gene expression data for 14,983 genes comparing E1 (mature fruit), E2 (shelf-life), and E3 (cold-stored fruit) against E4 (cold-stored plus shelf-life) under commercial thinning (TH) in MG and RP varieties were obtained from the Supplementary Information provided as Table S5 by Nilo-Poyanco et al. [10]. Transcriptomic information was incorporated as soft reaction-specific penalties during flux minimization, such that reactions associated with downregulated genes incurred higher penalties, whereas reactions associated with upregulated genes were assigned lower penalties. Reactions with mixed or ambiguous gene expression support were treated conservatively using intermediate weights, ensuring that no reaction was forcibly constrained or activated based solely on transcript levels.

### 2.7. Relative Growth Rate Assessment

Relative growth rate (RGR) defines fruit growth in terms of mass at a specific point in time. It was calculated as the natural logarithm of the mean fruit fresh weight of condition 2 minus that of condition 1, divided by the time elapsed between the two sampling points [40].

### 2.8. Protein Sequence Alignment

Protein sequences were downloaded from the Phytozome v12.1 portal (https://phytozome.jgi.doe.gov/pz/portal.html, accessed on 20 September 2017) and aligned using Clustal Omega [41] with the online default settings.

### 2.9. Statistical Analysis

Metabolite contents and phenotypic data were analyzed using principal component analysis (PCA) with InfoStat Statistical Software (version 2012). For phenotypic traits, statistical analyses were performed in R. Within each developmental stage, the effects of variety and thinning treatment were evaluated using two-way ANOVA (Variety × Thinning). Interaction effects were tested explicitly. When a treatment group was absent, interaction terms could not be estimated and therefore were not reported. For juiciness, comparisons between E2 and E4 within each variety × treatment combination were performed using Welch’s *t*-test to account for unequal variances and sample sizes.

## 3. Results

### 3.1. Fruit Development and Physicochemical Parameters Were Affected by Commercial Tree Thinning Treatment

**Figure 2:**
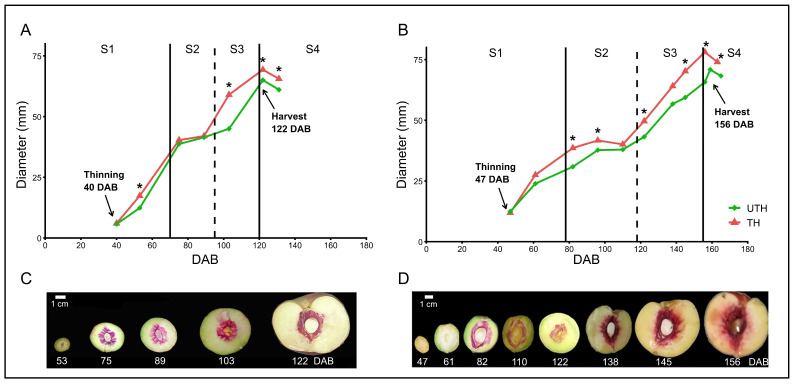
‘Magique’ (MG) and ‘Red Pearl’ (RP) fruit development in days after blooming (DAB) for thinned (TH, red) and unthinned (UTH, green) conditions. Fruit growth is shown for fruit collected during the 2015 growing season for MG (**A**) and RP (**B**), *n* ≥ 3. * Indicates significant differences between thinning conditions (Student’s *t*-test, *p*< 0.05). Developmental stages are indicated: S1—cell proliferation, S2—pit hardening, S3—cell enlargement, S4—postharvest. Fruits were harvested at 122 and 159 DAB for MG and RP, respectively. Lignin staining of MG fruit (**C**) and RP (**D**) fruit.

As a first step to understand the effect of thinning treatments to ameliorate CI in nectarines, the developmental process of MG and RP was characterized. MG is an early-harvest, white-flesh nectarine, whereas RP is a late-harvest, yellow-flesh nectarine [10]. To define the developmental stages of these varieties, fruit diameter and phloroglucinol-based lignin staining were measured along the growing season (Figure 2). The fruit growth curve followed a characteristic double-sigmoid pattern in both seasons (Figure 2A,B) which can be divided into four stages, S1 to S4 [42]. Commercial thinning was performed during S1 (40 DAB for MG and 47 DAB for RP). Lignin staining (magenta color, Figure 2C,D) was used to better determine the beginning of S2, which corresponds to stone lignification. S2 started between 53 and 75 DAB for MG, and between 61 and 82 DAB for RP. Phloroglucinol staining showed that the lignification process was precisely confined to the stone in MG throughout the entire developmental period, whereas in RP flesh staining could be seen at the transition between S3 and S4, and in S4. Reduced fruit diameter growth was also observed at S2 for RP.

Fruit was harvested at 122 DAB for MG and 156 DAB for RP in the trees subjected to commercial thinning (TH), and at 122 DAB for MG and 159 DAB for RP in the unthinned trees (UTH). These harvest timings reflect the early- and late-harvest characteristics of MG and RP varieties. Fruits from TH trees presented significantly higher diameters than UTH trees, with differences appearing at S3-S4 for MG and S2-S4 for RP (Tukey’s test, *p* < 0.05) (Figure 2A,B). Relative growth rate patterns showed differences during S3 between fruit RGR curves from TH and UTH trees, while fruit weight accumulation patterns were similar between treatments, though TH fruits maintained consistently higher weights (Figure S3). In MG, the change could be interpreted as a phase shift, whereas in RP the change was more related to curve amplitude.

**Figure 3:**
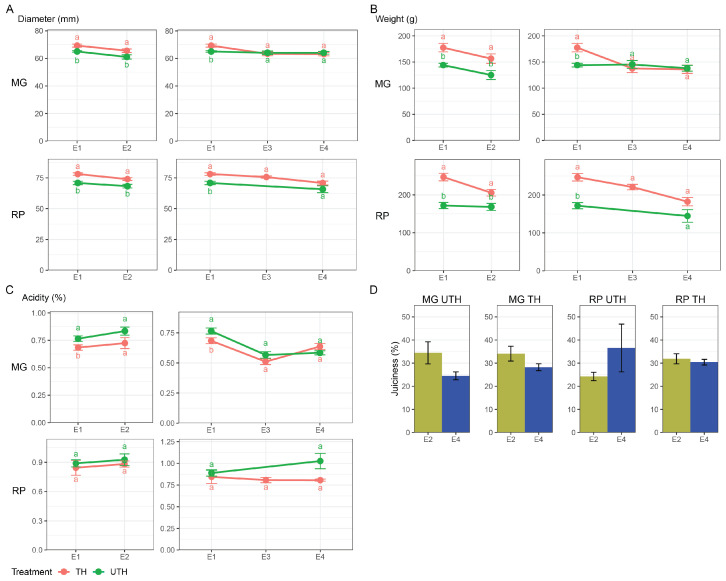
Postharvest characterization of MG and RP fruits from thinned (TH, red) and unthinned (UTH, green) trees. Comparison of (**A**) fruit diameter (mm), (**B**) fruit weight (g), (**C**) titratable acidity (%), and (**D**) juiciness (%) across postharvest stages E1, E2, E3, and E4. Lowercase letters indicate significant differences between treatments within the same stage.

To evaluate whether thinning effects depended on variety, a factorial analysis was performed (Table 1 and Table S1). Variety and thinning effects were generally independent, as significant variety × thinning interactions were only detected for fresh weight at E1 and acidity at E4. Fruits from TH trees of both varieties displayed significantly larger diameters and higher fresh weights compared with UTH trees at harvest (E1) and after regular ripening (E2; Figure 3), though the weight effect of thinning was more pronounced in RP fruits at E1 (significant interaction, *p* = 0.009, Table 1). MG fruits from UTH trees were more acidic than TH fruits at E1, with no significant differences at any other stage. At E4, a significant variety-by-thinning interaction (*p* = 0.002) indicated a thinning effect on acidity that depended on variety (Table 1).

Juiciness showed no significant variety × thinning interactions at either evaluated stage: MG fruit juiciness followed a similar trend in both UTH and TH trees, with higher values at E2. For RP, UTH fruits at E4 displayed a trend toward higher juiciness than E2 fruits, whereas in TH fruits no difference was observed.

### 3.2. The Effects of Cold Storage on Sugar and Organic Acid Contents Were Variety-Specific

**Figure 4:**
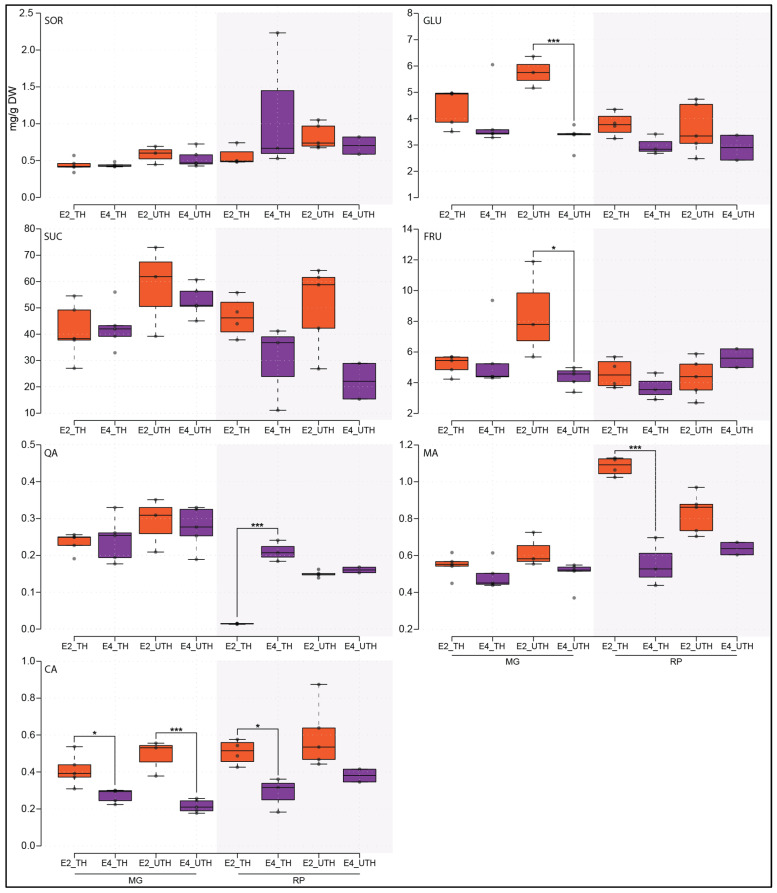
Box plot representation of metabolite concentrations in MG and RP fruits from unthinned (UTH) and thinned (TH) trees at E2 (shelf-life, orange) and E4 (cold-stored plus shelf-life, purple) postharvest treatments. Abbreviations: SOR, sorbitol; GLU, glucose; FRU, fructose; SUC, sucrose; MA, malic acid; CA, citric acid; QA, quinic acid. Asterisks represent significant difference between E2 and E4 stage comparison within the same variety (*n* ≥ 2, Student’s *t*-test, * = *p* < 0.05, *** = *p* < 0.005).

Soluble sugars and organic acid contents from fruit mesocarp polar extracts were analyzed to detect changes in metabolite composition associated with various postharvest conditions and crop load treatments. Four sugars (sorbitol, glucose, sucrose, and fructose) and three organic acids (quinic, malic, and citric acids) were measured in MG and RP (Table S2). In fruit from UTH MG trees, glucose, fructose and citric acid concentrations were significantly lower in fruit ripened after cold storage (E4) compared to regularly ripened (E2) fruit (Figure 4, Student’s *t*-test, *p* < 0.05). In fruit from TH MG trees, when comparing E2 and E4 stages, a significant difference was found in citric acid content only (Figure 4). Thus, for the MG variety, TH tree fruits displayed similar concentrations of metabolites between E2 and E4, indicating that low crop loads for this variety might be beneficial when MG fruits must be stored at low temperatures.

For the RP variety, fruit from UTH trees showed no metabolites with significantly higher contents in E2 than in E4 (Figure 4), whereas in fruit from TH trees, citric and malic acid contents were significantly higher in E2 than in E4 (Figure 4). In turn, quinic acid content was significantly higher at E4 compared to E2 in fruit from TH trees, with an increase of approximately 14-fold (Figure 4). Thus, for RP, crop loading management did not help preserve organic acid accumulation at E4 relative to E2.

### 3.3. Postharvest Condition Was the Main Factor Differentiating the Assessed Samples

**Figure 5:**
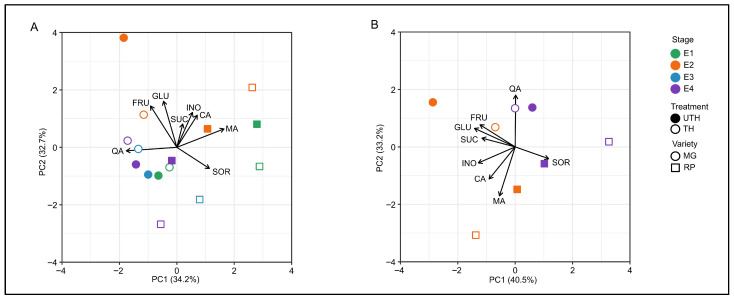
Principal component analysis (PCA) of sugar and organic acid data from MG and RP fruit subjected to different postharvest conditions. (**A**) E1 (green), E2 (orange), E3 (blue), and E4 (purple). (**B**) E2 and E4 only. Principal component 1 (PC1) is displayed on the X-axis and PC2 on the Y-axis. The solid symbols correspond to fruit from unthinned trees (UTH), while the open symbols correspond to fruit from thinned trees (TH). Circles represent the MG variety, and squares represent the RP variety. Arrow length reflects the contribution of each variable to the displayed principal components. The variance explained by each PC (%) is given in parentheses. E3 unthinned tree data for RP were not available.

Principal component analysis (PCA) was used to cluster the postharvest stages and different thinning treatments of the two varieties evaluated based on their metabolic composition (Figure 5). Two principal components (PCs) explained 66.9% of the overall variance. PC1 (34.2% variance) separated MG (represented by circles) from RP (represented by squares). The metabolites that most contributed to the positive side of PC1, and were more abundant in MG, were quinic acid, fructose and glucose. The metabolites that were more abundant in RP (on the negative side of PC1) were sorbitol and malic acid (Figure 5A). PC2 mainly separated regularly ripened (E2) fruit from all the other conditions, regardless of variety or preharvest treatment. Concerning the chilling-sensitive variety RP, E1 and E2 were grouped apart from E3 and E4, whereas for the chilling-resistant variety MG this pattern was not found. When accounting for thinning, the only major effect was related to regular ripe (E2) fruit in the MG variety, where UTH-derived fruit departed from all other conditions.

When the PCA was performed using metabolite contents of the E2 and E4 conditions only, the two principal components explained 73.7% of the overall variance. PC1 (40.5% variance) separated MG from RP (Figure 5B), with sugars being the main driver of this separation. PC2 (33.2% variance) separated E2 from E4 with the exception of TH MG E2, which clustered with E4 samples. Malic and citric acids tended to have higher contents in E2, while quinic acid tended to have higher contents in E4 (Figure 5B).

### 3.4. Differential Phenylpropanoid Metabolism Between ‘Magique’ and ‘Red Pearl’ During Regular Ripening and Chilling Ripening

A genome-scale metabolic model (GSM) [43] of peach fruit was developed to mechanistically investigate metabolic processes underlying differences in chilling injury susceptibility between MG and RP varieties. The metabolic network was initially assembled from a manually curated reaction list (Table S3) and loaded programmatically using COBRApy (Version 0.30.0). The final functional model comprises 185 reactions, 150 metabolites, 410 genes, and 9 major metabolic processes.

The model constitutes a feasible, mass-balanced, and internally consistent representation of fruit mesocarp metabolism operating under a maintenance-only regime, enforced via non-growth-associated maintenance (NGAM) ATP demand. Consistent with the autonomous nature of mature fruit ripening, the model does not maximize biomass production but instead optimizes a custom ripening quality objective (RIPENING_QUALITY_SINK, RQS), which enforces the simultaneous production of four key ripening-associated traits: chlorogenate (phenylpropanoid metabolism), cyanidin (anthocyanin biosynthesis), ethylene (climacteric hormone), and galacturonate (pectin depolymerization).

To integrate transcriptomic information, we computed the minimum-flux solution capable of sustaining the RQS while incorporating gene expression contrasts as soft reaction-specific penalties. This analysis identified a limited set of eight reactions that must remain metabolically active despite being transcriptionally disfavored during the transition from mature (E1) to ripe fruit (E2) or during post-chilling ripening (E3 to E4). These eight reactions represent candidate regulatory control points, where physiological demands associated with ripening quality override transcriptional trends (Table 2).

**Figure 6:**
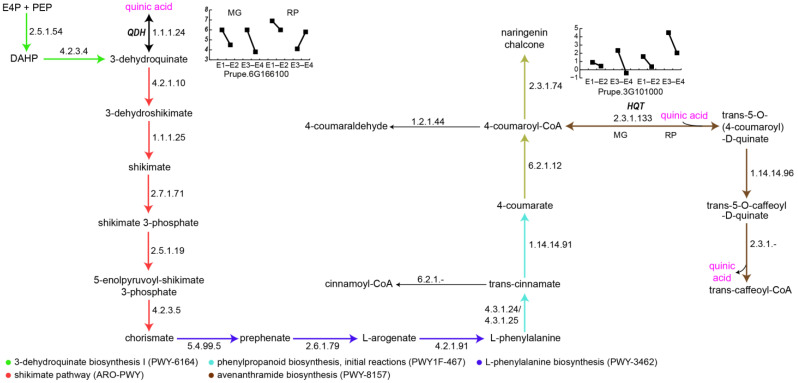
Shikimate and phenylpropanoid pathways highlighting candidate regulatory nodes identified through genome-scale metabolic modeling. Pathway segments are color-coded by metabolic subsystem as indicated in the legend (bottom left). Quinic acid (magenta) appears at multiple branch points, underscoring its central role in pathway flux. Inset graphs show log2-transformed counts per million (CPM) for two key enzyme-encoding genes, quinate dehydrogenase (QDH, Prupe.6G166100) and hydroxycinnamoyl-CoA quinate transferase (HQT, Prupe.3G101000), across ripening stages E1-E2 and E3-E4 in MG and RP varieties. EC numbers label individual reactions.

Among the identified regulatory levers, hydroxycinnamoyl-CoA quinate transferase (HQT, EC 2.3.1.133) and quinate dehydrogenase (QDH, EC 1.1.1.24) emerged as consistent determinants of ripening-associated phenylpropanoid flux (Table 2, Figure 6). HQT carried a positive flux in all contrasts and in both varieties, indicating that the model consistently routed carbon toward p-coumaroylquinate formation to support chlorogenate production. In parallel, transcriptomic data showed a downregulation of its expression during ripening (Figure 6, Prupe3G101000). Together, these data indicate that HQT remained required to achieve the RQS objective despite transcriptional downregulation, identifying it as a candidate regulatory lever linking shikimate-derived carbon partitioning to ripening-associated phenylpropanoid demand.

Regarding QDH, based on its reaction stoichiometry (Table 2), a negative flux indicates net conversion of quinate to dehydroquinate coupled to NADH regeneration, supporting the identification of QDH as a metabolic lever contributing to redox balance and shikimate pathway flux during ripening. Notably, this lever behavior was absent during the RP E3-to-E4 transition, which coincides with the pronounced accumulation of quinic acid observed in chilled RP fruit (Figure 4), indicating a condition-specific relaxation of quinate utilization. At the transcriptome level, the gene encoding QDH (Prupe6G166100; Figure S5) was downregulated during ripening in MG E1 to E2, MG E3 to E4, and RP E1 to E2, while exhibiting the opposite expression pattern in RP E3 to E4 (Figure 6).

### 3.5. Thinning Drives Divergent Phenylpropanoid and Quinate Accumulation Patterns in MG and RP Peach Fruit at the E1 Stage

**Figure 7:**
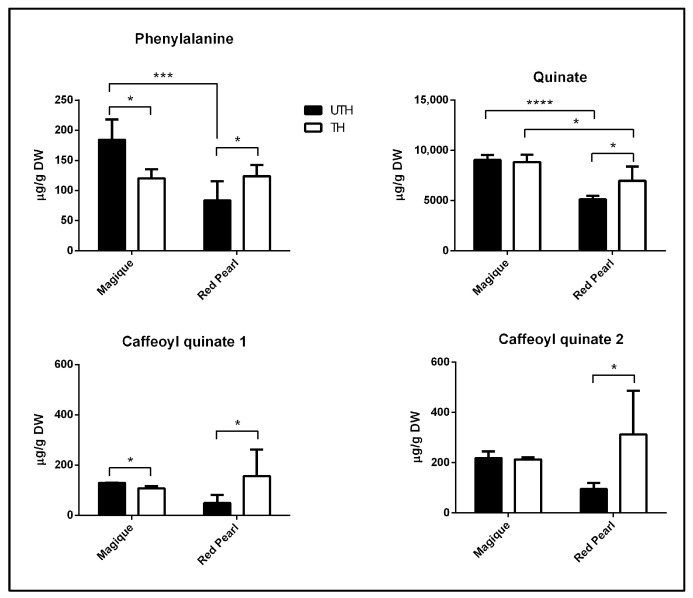
Contents of four phenylpropanoid pathway intermediates, determined using 1H-NMR profiling, in fruits from unthinned (UTH) and thinned (TH) trees in MG and RP at harvest (E1). Bars represent the average of three biological replicates (*n* = 3); error bars represent standard deviation. Asterisks indicate significant differences between thinning treatments or varieties. To compare between varieties, a *t*-test was used (*p*< 0.05). To compare between thinning treatments, a two-way ANOVA followed by Sidak’s multiple comparison test was used (* *p*< 0.05; *** *p*< 0.0005; **** *p*< 0.00005).

The thinning effect at the E1 stage on additional phenylpropanoid pathway metabolites was assessed using 1H-NMR-based targeted analyses. Phenylalanine content in MG was significantly higher at the E1 stage in UTH fruits compared with TH fruits, while in RP the pattern was the opposite (Figure 7). Quinate concentration in MG fruit was already high at the E1 stage while RP reached the E1 stage with nearly half of this value (Figure 7, Table S2). Thinning treatment stimulated quinate accumulation in RP, with no apparent effect in MG. Finally, two caffeoyl quinate compounds, intermediates of the phenylpropanoid biosynthesis pathway, were significantly higher in RP fruits from TH trees than from UTH trees (Figure 7), a pattern not found for MG.

## 4. Discussion

Thinning is an agronomic practice that alters the source–sink ratio, affecting plant photosynthetic rates and reducing fruit–fruit competition, ultimately increasing final fruit size [26,44,45]. Accordingly, both MG and RP peach varieties subjected to commercial thinning (TH) treatment displayed increased fruit diameter (Figure 2 and Figure 3). However, their developmental patterns differed, with RP TH already showing increased size compared to the UTH tree fruits at S2, whereas MG TH fruits displayed greater size only at S3 (Figure 2). Peach fruit growth follows a double-sigmoid curve with four main stages [46], with the S2 stage displaying the least amount of growth, possibly due to the deviation of assimilates and hormones toward endocarp lignification and anthocyanin biosynthesis. MG, an early-harvest variety, displayed a more compact double-sigmoid curve than the late-harvest Red Pearl (RP; Figure 2) with a shorter S2 stage, a typical difference between early and late varieties [47,48].

Peach fruit growth rates can also be assessed by comparing relative growth rate (RGR) curves, which account for the fruit mass increase at a specific point in time. Peach fruit RGR is characterized by an initial plateau, followed by a decrease and a second peak, which is always lower than the initial plateau [40]. Both MG and RP displayed this pattern but with marked differences. The more compact double-sigmoid curve in MG implied an RGR curve with much more abrupt changes than that observed in RP (Figure S3). In addition, in MG the S2 phase was shifted to an earlier time in TH fruits compared with UTH fruits, which can have negative consequences for fruit stone lignification (see below). In RP, the slope of TH fruit was more pronounced from the initial plateau into S2 than in UTH fruit (Figure S3). This could explain why RP fruits from TH trees displayed a difference in size as early as the S2 stage, i.e., in RP fruits from TH trees, fruits were already at a higher initial plateau than UTH trees.

The stone lignification phase (S2) can be advanced and/or very short in thinned trees [45]. It seems that in the MG variety, endocarp hardening occurs in parallel with fruit growth, which requires resource sharing between both metabolic pathways. We have observed stone breakage in MG fruits from TH trees (Figure S4), which could be due to the disproportionate mesocarp radial tensile force exerted on the stone or to deficient lignification in this variety [49,50]. Therefore, it is expected that stone lignification is reinforced in thinned trees to counteract these tensile forces. Among the resources required to generate lignin are chlorogenic acids (CGAs) [51], which are among the most abundant phenolics in peach mesocarp [52]. CGAs are soluble esters formed by the conjugation of trans-cinnamic acids and quinic acid, and include 5-O-caffeoylquinic acid, caffeoyl acids, p-coumaroylquinic acids, and feruloylquinic acids [51]. Based on the higher amounts of CGAs in RP fruits from TH trees (Figure 7), it is plausible that RP reinforces its endocarp lignification in commercially thinned trees by increasing CGA availability, whereas this pattern was not observed in MG.

The accumulation pattern of quinic acid (Figure 4) and CGAs (Figure 7) was very similar between TH and UTH fruits, and between ripe (E2) and post-chilling ripe (E4) in MG fruits, indicating that quinic acid and other phenylpropanoid abundances are robust to external stimuli in this chilling tolerant variety. Organic acid and CGA levels in RP fruits showed the opposite trend, being susceptible to both thinning treatment as well as cold storage. The recent literature has explored the role of exogenous treatments, such as melatonin treatment, in modulating the phenylpropanoid pathway through DNA methylation mechanisms to alleviate mesocarp browning due to chilling injury [53]. Melatonin-treated fruits presented increased levels of phenolic compounds such as chlorogenic acid and caffeoyl acids, effectively mitigating chilling-induced browning in peach fruit. Quinic acid and its derivatives have been reported to be related to biotic and abiotic stress tolerance, including pathogen attacks, low or high temperatures, UV light, drugs, heavy metals, saline stress and peach fruit chilling injury [28,52,54,55,56], which may be related to their role as precursors of specialized metabolites involved in stress tolerance [57,58,59]. Previous studies have shown that elevated expression levels of genes related to antioxidant systems at harvest and during cold storage—and the biosynthesis of metabolites with antioxidant activity such as carotenoids, flavonoids and proanthocyanins—correlate with tolerance [60]. In contrast, other transcriptomic and proteomic studies concluded that metabolic rearrangements may occur prior to cold exposure in chilling-tolerant varieties [60,61]. This may be the case for MG, a chilling-tolerant variety, whereas in RP, a chilling-sensitive variety, the cold tolerance response is triggered only after cold exposure.

Consistent with these observations, the genome-scale metabolic model identified quinate-centered reactions as key metabolic control points differentiating chilling-tolerant and chilling-sensitive fruit. In particular, quinate dehydrogenase (QDH) and hydroxycinnamoyl-CoA quinate transferase (HQT) emerged as regulatory levers whose fluxes were required to sustain ripening-associated phenylpropanoid demand, even when transcript levels were reduced. Model simulations predicted a sustained net utilization of quinate during ripening in MG and RP under non-chilling conditions, whereas this behavior was relaxed specifically during post-chilling ripening in RP (E3 to E4). This condition-specific loss of quinate utilization capacity provides a mechanistic framework consistent with the marked accumulation of quinic acid observed in chilled RP fruit and supports the idea that chilling sensitivity in RP is associated with an impaired ability to redirect quinate toward downstream phenylpropanoid and antioxidant pathways. This is also consistent with the red pigment accumulation (mesocarp bleeding) seen in chilling-injured RP fruit (E4, panels A and B, Figure S4) that could be related to impaired anthocyanin accumulation [62].

Phenylpropanoid accumulation in fruits can be regulated by vacuole transporters and by the abundance of the enzymes involved in its metabolism [63,64]. Several of the genes encoding these metabolic enzymes have well-documented transcriptional regulators, such as myeloblastosis (MYB) transcription factors (TFs) [65,66,67]. MYB proteins can act in concert with basic Helix Loop Helix (bHLH) and WD40 regulators, forming the MBW complex, which binds to the promoters of their target genes [67,68]. In addition to their role in regulating phenylpropanoids abundance, TFs from the MBW complex have also been reported as being modulated by temperature [69,70]. Given the contrasting expression pattern of quinate dehydrogenase between MG and RP varieties observed in this work, and its role as key enzyme for quinate accumulation [71], a promising next step would be the characterization of MBW complex TFs that regulate the quinate dehydrogenase gene.

RP is more susceptible to chilling stress than MG and typically exhibits pulp wooliness/mealiness and bleeding. In our study, we observed bleeding in RP fruits in response to chilling treatment (Figure S4). The normal juiciness observed in RP E4 is not surprising, as this trait has previously been shown to have low heritability and to be strongly influenced by environmental factors, including preharvest conditions [60].

Ripe fruit after cold storage (E4) displayed a metabolically disturbed profile that could be related to the fruit’s response to cold stress, which can lead to a rearrangement in metabolic pathways [54] and depends on enzyme stability and catalytic activity to restore homeostasis and normal metabolic fluxes [72]. In addition, the tolerance mechanisms of each variety and the duration of cold exposure modulate the fruit’s response [54]. Fruit acidity depends on both the content and composition of organic acids, and the balance between sugars and acids is a key determinant of fruit taste [73,74]. In this work, citric acid content decreased in both varieties in response to cold treatment regardless of thinning treatment (Figure 4). In contrast, a decrease in malic acid content was observed in RP TH only. These findings suggest that these organic acids are rapidly metabolized during and after cold storage, which probably affects the organoleptic properties of the ready-to-eat fruit. RP fruits from TH trees appear to be the most affected, as the relative proportion of the three analyzed organic acids changed markedly after cold treatment.

The MG and RP varieties evaluated in this work must meet consumers’ high quality standards after the extended cold storage that is required for export to distant markets. Under our conditions, the main metabolic difference observed between them was the high constitutive level of quinic acid in MG, which may contribute to its higher tolerance to cold stress. In RP, quinic acid content increased in response to cold exposure; however, fruit bleeding was still observed in the mesocarp, suggesting that high constitutive levels of quinic acid might be required prior to ripening to provide protection against chilling injury. While these findings support a mechanistic association between quinate metabolism and chilling tolerance, further functional studies will be required to establish a direct causal relationship.

We also observed that thinning improved fruit size in both varieties but also induced stone cracking in MG (Figure S4). This is likely due to the diversion of carbon skeletons from endocarp lignification toward mesocarp growth, suggesting that commercial thinning practices may be disadvantageous for early-season varieties.

## 5. Conclusions

In this work, we show that although the phenylpropanoid pathway is traditionally considered a secondary metabolic pathway, it might play a central role in peach fruit physiology and is strongly influenced by both pre- and postharvest practices, ultimately affecting final fruit quality [30]. Thinning, as a pre-harvest strategy, had a differential effect on early and late nectarine varieties. This could be related to different modulation of fruit phenylpropanoid metabolism, associated with how the metabolic resources generated by this pathway are used when fruit development and ripening spans a relatively short (early) versus a prolonged (late) pre-harvest period. Thus, thinning as a pre-harvest strategy should be performed in a way that integrates the developmental window of a given variety.

Post-harvest cold storage impact on fruit quality was also associated with the phenylpropanoid-derived metabolite pool and could therefore be minimized if the fruit has been able to perform a proper quinic acid metabolism prior to ripening. The main metabolic difference observed between MG and RP was the high constitutive level of quinic acid in MG, which may contribute to its higher tolerance to cold stress. Genome-scale metabolic modeling helped identify quinate dehydrogenase (QDH) and hydroxycinnamoyl-CoA quinate transferase (HQT) as key regulatory control points differentiating chilling-tolerant and chilling-sensitive fruit. Expression profile of these genes predicted an impaired capacity to redirect quinate toward downstream phenylpropanoid and antioxidant pathways specifically during post-chilling ripening in RP. While these findings support a mechanistic association between quinate metabolism and chilling tolerance, further functional studies will be required to establish a direct causal relationship.

## Figures and Tables

**Table 1:** Two-way ANOVA results (variety × thinning).

Stage	Variety	Thinning	Interaction
DIAMETER			
E1	3.80 × 10^−10^	7.18 × 10^−7^	1.62 × 10^−1^
E2	3.73 × 10^−6^	7.43 × 10^−4^	6.71 × 10^−1^
E3	1.30 × 10^−10^	6.53 × 10^−1^	NA
E4	2.74 × 10^−2^	9.44 × 10^−1^	1.45 × 10^−1^
WEIGHT			
E1	6.29 × 10^−9^	3.31 × 10^−9^	9.47 × 10^−3^
E2	2.01 × 10^−5^	4.49 × 10^−4^	7.55 × 10^−1^
E3	2.09 × 10^−11^	4.84 × 10^−1^	NA
E4	4.12 × 10^−2^	6.62 × 10^−1^	1.22 × 10^−1^
ACIDITY			
E1	4.10 × 10^−3^	1.82 × 10^−1^	7.07 × 10^−1^
E2	9.06 × 10^−3^	9.60 × 10^−2^	5.04 × 10^−1^
E3	1.06 × 10^−10^	1.50 × 10^−1^	NA
E4	6.33 × 10^−9^	6.64 × 10^−1^	1.91 × 10^−3^
JUICINESS			
E2	3.90 × 10^−2^	2.99 × 10^−1^	2.24 × 10^−1^
E4	4.10 × 10^−2^	3.43 × 10^−1^	1.50 × 10^−1^

NA indicates interaction not estimable due to missing treatment group.

**Table 2:** Candidate regulatory levers for peach fruit ripening by metabolic modeling in MG and RP nectarine fruit.

		MG		RP	
	REACTION	E1vsE2	E3vsE4	E1vsE2	E3vsE4
ACO_m	cit_m <=> isocit_m	NA	NA	NA	0.3 (-->)
CAS_c	cys_c + hcn_c --> cyanoala_c + h2s_c + h_c	1	NA	1	1
EPSPS_c	pep_c + s3p_c --> epsp_c + pi_c	1	NA	NA	NA
FUM_m	fum_m + h2o_m <=> mal_m	NA	NA	NA	0.3 (-->)
GR_c	gssg_c + h_c + nadph_c --> 2.0 gsh_c + nadp_c	NA	NA	NA	1
HQT_c	pcoumcoa_c + quinate_c <=> CoAc + pcoumaroylqnt_c	1 (-->)	1 (-->)	1 (-->)	1 (-->)
PPA_AT_c	glu_c + prephenate_c --> akg_c + arogenate_c	NA	NA	1	NA
QDH_c	dhq_c + h_c + nadh_c <=> nad_c + quinate_c	2 (<--)	2 (<--)	2 (<--)	NA

Listed reactions must remain metabolically active to sustain the ripening quality objective (RQS) across two developmental transi-tions: regular ripening (E1 vs. E2) and post-chilling ripening (E3 vs. E4). Values indicate normalized flux magnitude; NA denotes reactions not identified as regulatory levers in that condition. Arrows are shown inside parentheses (-->, <--) to indicate the predicted flux direction when the reactions are reversible; ‘_c’—cytosol, ‘_m’—mitochondria; E1—harvest stage, E2—shelf-life, E3—cold-stored, E4- cold-stored plus shelf-life fruit; akg—ketoglutarate, cyanoala—cyanoalanine, cit—citrate, cys—cysteine, dhq—dehydroquinate, epsp—5-enolpyruvylshikimate 3-phosphate, glu—glutamate, gsh—glutathione, gssg—glutathione disulfide, hcn—hydrogen cyanide, h2s—hydrogen sulfide, iso—isocitrate, mal—malate, pep—phosphoenolpyruvate, pcoumaroylqnt—p-Coumaroylquinate, pcoumcoa—p-Coumaroyl-CoA, s3p—shikimate 3-phosphate.

## Data Availability

The original contributions presented in this study are included in the article and Supplementary Materials. Further inquiries can be directed to the corresponding author.

## Supplementary Materials

The following supporting information can be downloaded at: https://www.mdpi.com/article/10.3390/metabo16030191/s1, Table S1: Postharvest evaluation of ‘Magique’ and ‘Red Pearl’ fruit; Table S2: Liquid chromatography data from sugars and organic acids from MG and RP; Table S3: Genes and reaction templates used for metabolic modeling; Figure S1: HPAEC-PAD chromatogram of sugars in ‘Red Pearl’ variety; Figure S2: HPLC-DAD chromatogram of organic acids in ‘Red Pearl’ variety; Figure S3: Fruit growth rate analysis; Figure S4: Phenotypic analysis of mesocarp and endocarp during fruit development and ripening in MG and RP; Figure S5: Bioinformatics determination of quinate dehydrogenases from *Prunus persica*.

## Abbreviations

The following abbreviations are used in this manuscript:

Ant	anthocyanins
CA	citric acid
CGA	Chlorogenic acid
CI	Chilling injury
CPM	counts per million
DAB	days after full bloom
FBA	flux balance analysis
FBL	Fruit flesh bleeding
FRU	fructose
GLU	glucose
bHLH	basic Helix Loop Helix transcription factor
MA	malic acid
MG	Magique
MYB	myeloblastosis transcription factor
QA	quinic acid
RGR	relative growth rate
RP	Red Pearl
RQS	ripening-quality-sink
SOR	sorbitol
SUC	sucrose
TF	transcription factor
TH	Commercial thinning
TSS	Total soluble solids
UTH	Unthinning
