# Supplementary figures and images for: The Phenylpropanoid Pathway Is a Central Roundabout in Peach Fruit Pre- and Postharvest Physiology
Source: Metabolites. 2026 Mar 12;16(3):191. doi: 10.3390/metabo16030191 (PMC13028256; doi:10.3390/metabo16030191)

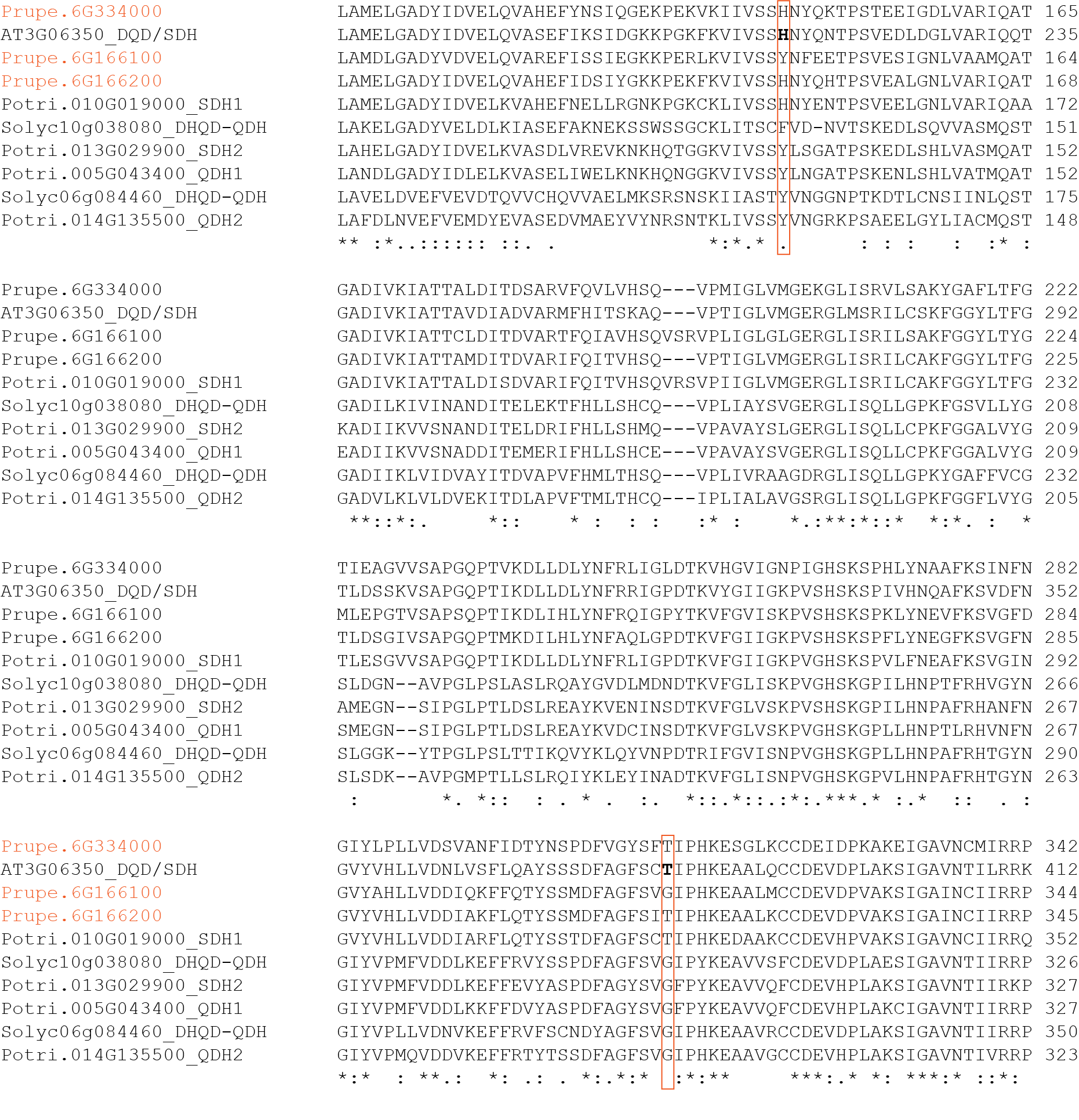

Supplement: Supplementary file 1 [file metabolites-16-00191-s001.zip › FigS5.png]

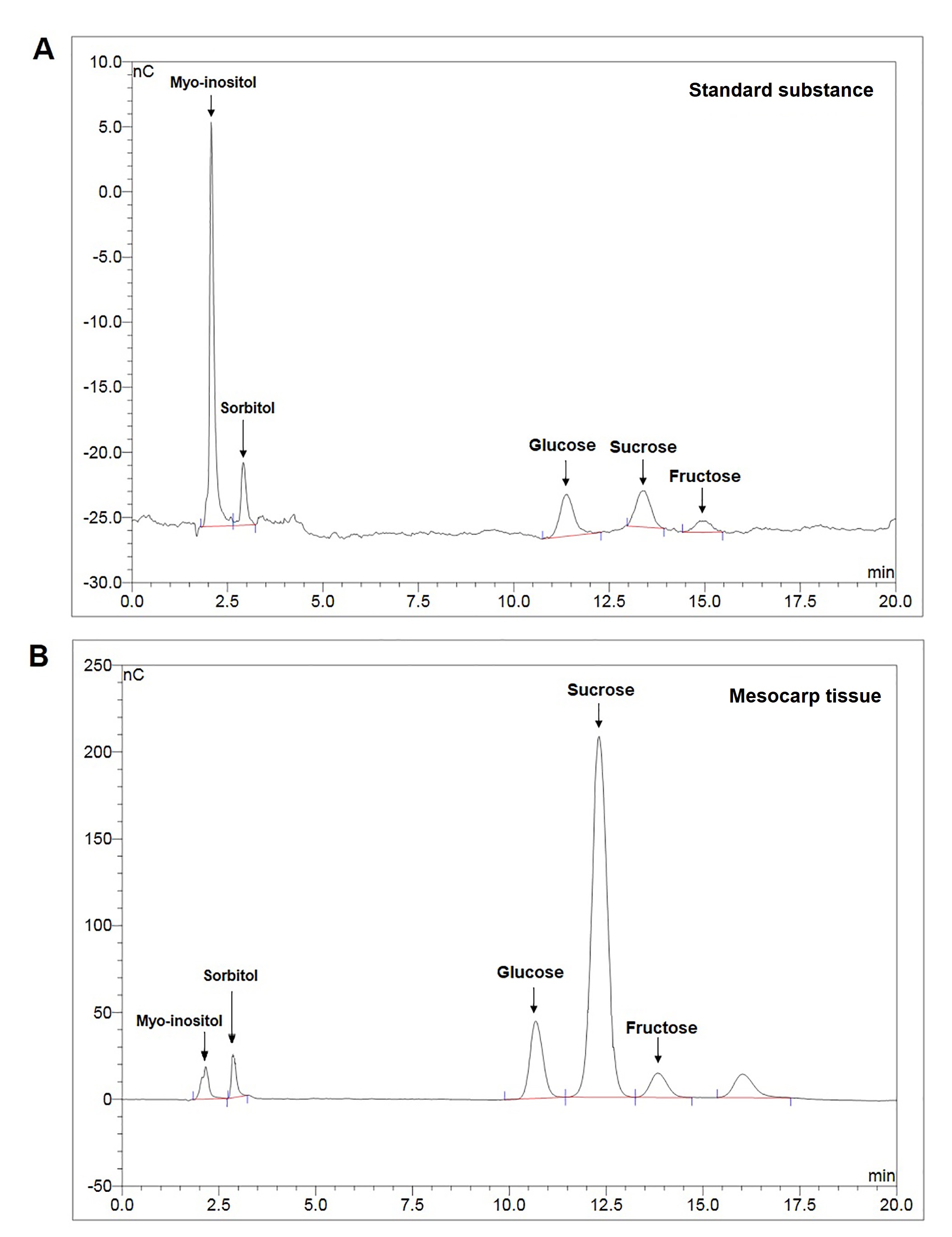

Supplement: Supplementary file 1 [file metabolites-16-00191-s001.zip › FigS1.jpg]

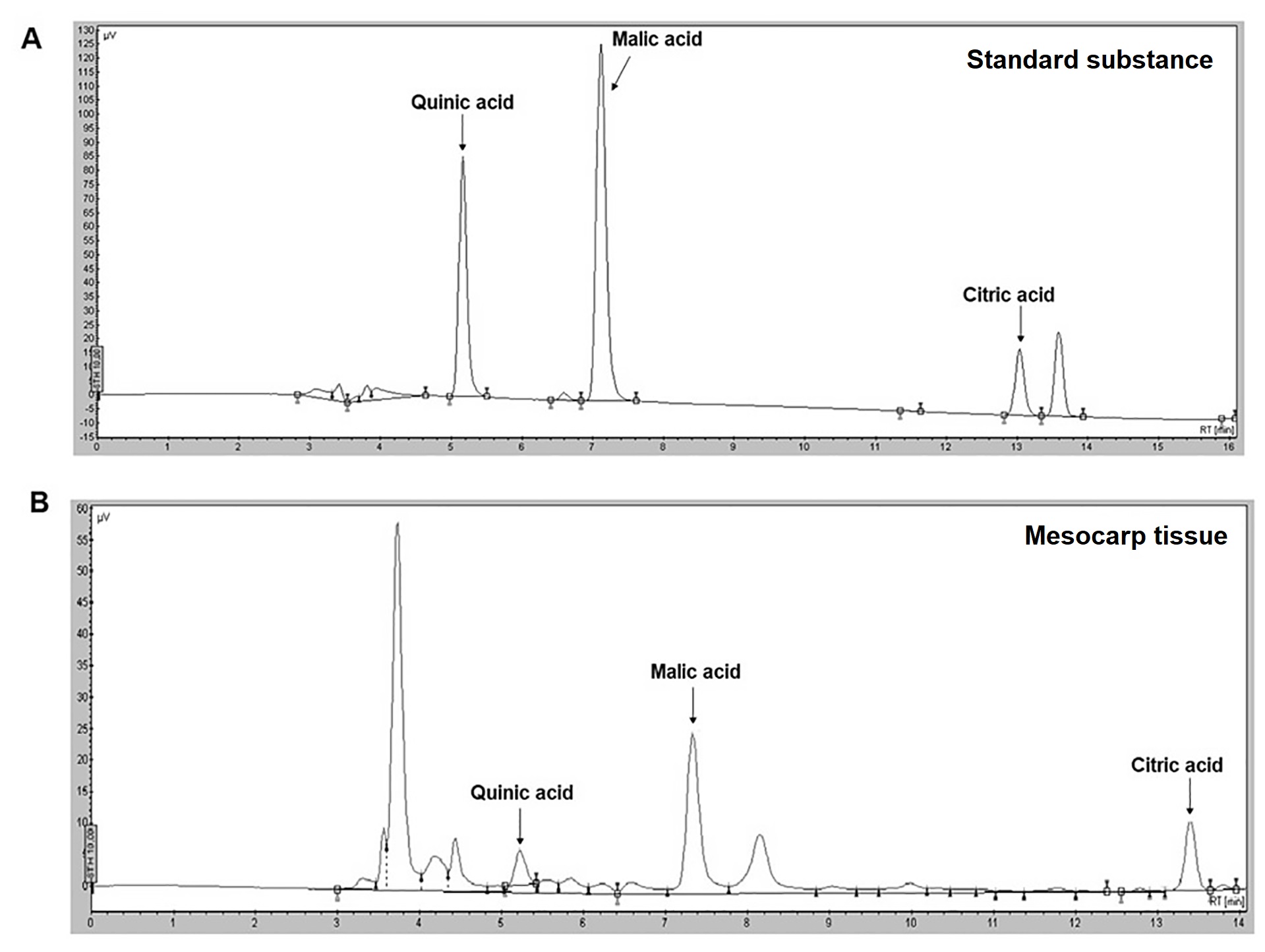

Supplement: Supplementary file 1 [file metabolites-16-00191-s001.zip › FigS2.jpg]

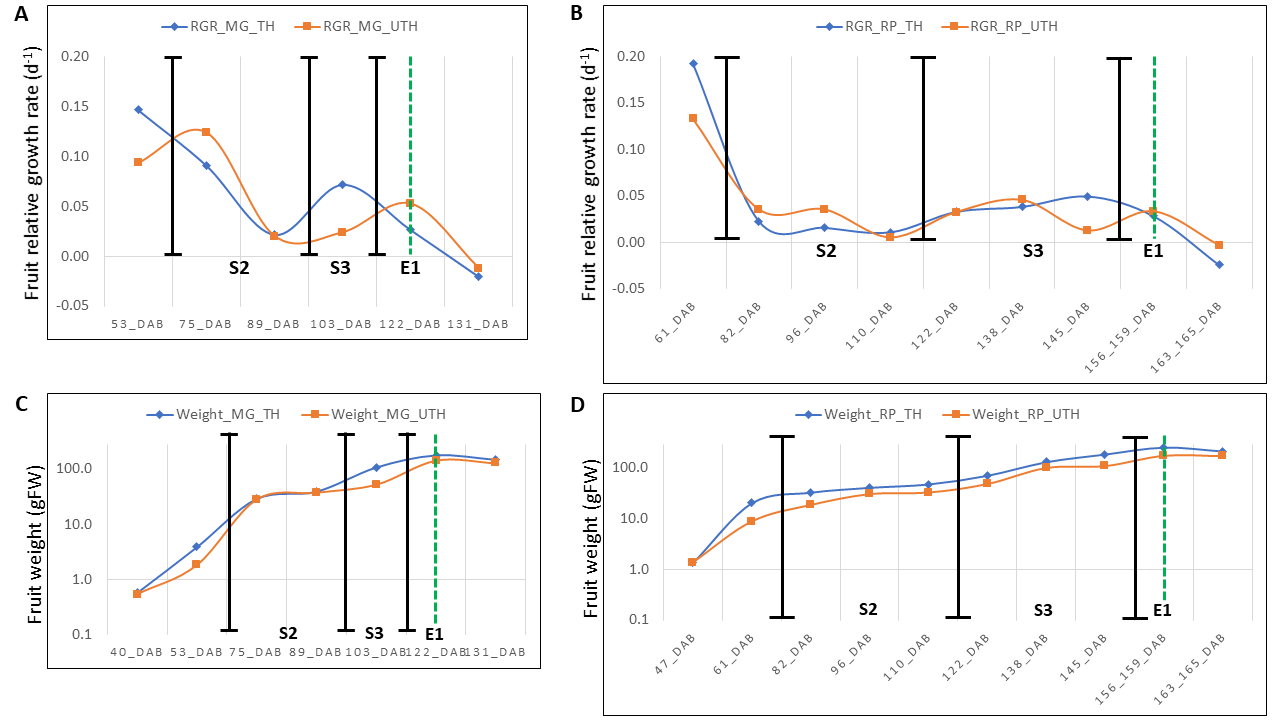

Supplement: Supplementary file 1 [file metabolites-16-00191-s001.zip › FigS3.tif]

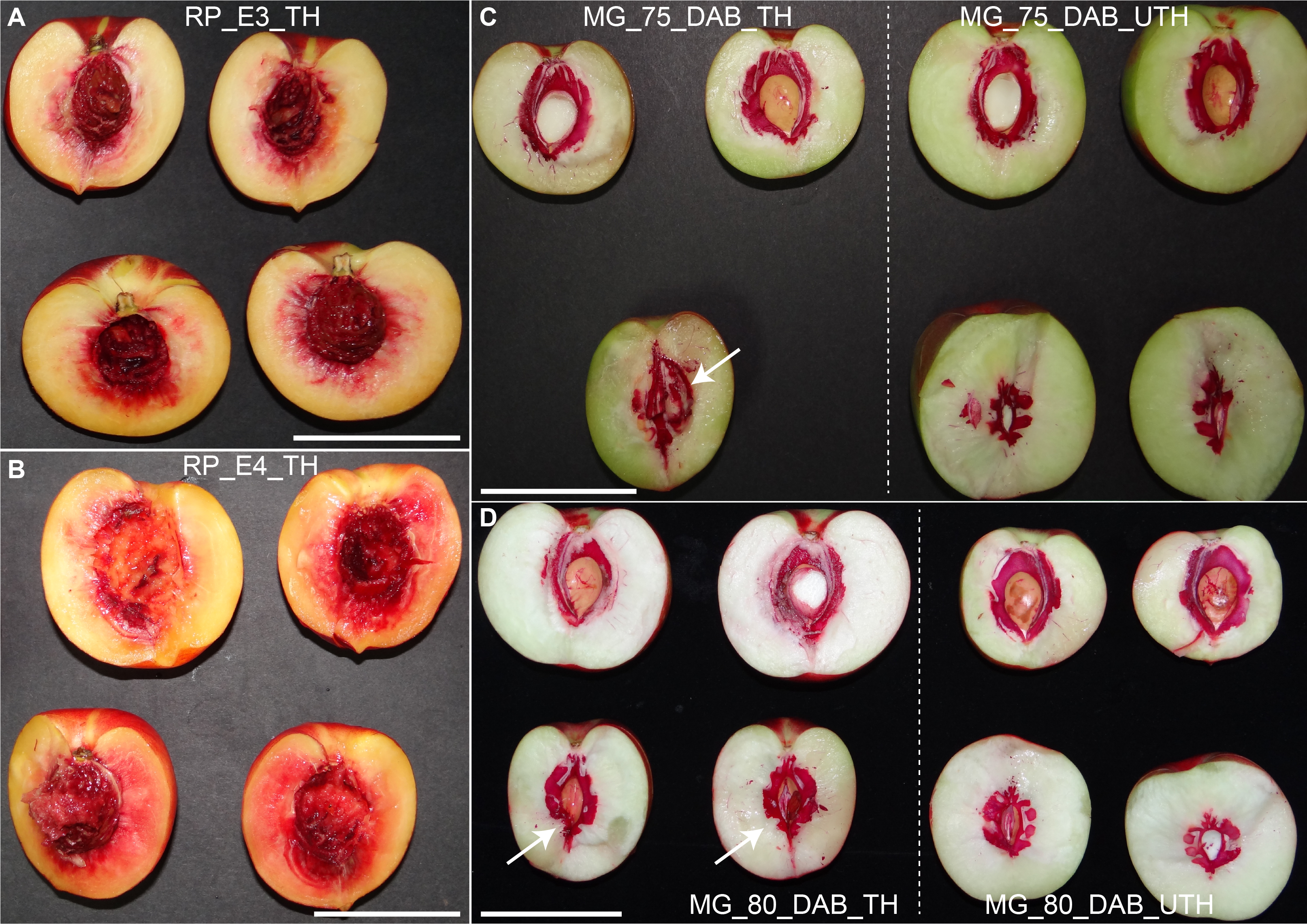

Supplement: Supplementary file 1 [file metabolites-16-00191-s001.zip › FigS4.png]
